# Preloaded combination nicotine replacement therapy for smoking cessation in Kazakhstan: A randomized controlled trial study protocol

**DOI:** 10.1371/journal.pone.0292490

**Published:** 2023-11-27

**Authors:** Chethan Purushothama, Byron Lawrence Crape, Valentina Stolyarov, Altynshash Jaxybayeva, Philip la Fleur, Jeby Jose Olickal

**Affiliations:** 1 School of sciences & Humanities, Nazarbayev University, Nur-Sultan city, Republic of Kazakhstan; 2 Nazarbayev University School of Medicine, Nur-Sultan city, Republic of Kazakhstan; 3 Astana Medical University, Astana, Republic of Kazakhstan; 4 Department of Medicine, Nazarbayev University School of Medicine, Astana, Republic of Kazakhstan; 5 Department of Public Health, Amrita Institute of Medical Sciences, Kochi, Kerala, India; Università degli Studi di Ferrara, ITALY

## Abstract

**Background:**

Tobacco use is a major cause of premature death and disease in Kazakhstan, with over 22,500 deaths per year. Although efforts have been made to control tobacco use, smoking-related deaths have continued to increase. One strategy to help smokers quit is to use nicotine replacement therapy (NRT), with combination NRT resulting in higher long-term quit rates than a single form of NRT. A study aims to determine the effectiveness of preloaded combination NRT on smoking cessation, the change in health-related quality of life due to smoking cessation, and explore treatment adherence perceptions.

**Methods and analysis:**

The study will be conducted as a randomized, single-blind superiority trial, with 100 participants in each arm. The trial will be carried out at the National Research Cardiac Surgery Center, Astana, Kazakhstan, and will recruit current smokers aged 18 years and above with a motivation to quit. Participants will be randomly allocated to either the intervention group or the control group. The former will receive preloaded combination NRT, while the latter will receive fast-acting NRT alone. The primary outcome measure will be sustained abstinence from smoking after six months. Secondary outcome measures will include health-related quality of life and adherence to the treatment.

**Discussion:**

The study may gather further evidence that a combination NRT is more efficient than a fast-acting NRT alone. The findings of this study may help to improve tobacco cessation strategies in Kazakhstan and other countries with high smoking prevalence rates.

**Trial registration number:**

NCT05484505.

## Introduction

Tobacco use is the world’s leading preventable cause of premature death and disease-causing over 5 million deaths annually around the globe. The proportion of deaths due to smoking in Kazakhstan in 2004 was 24%, twice the worldwide rate (12.0%) and about 50% higher than the rates in Eurasia and the Russian Federation as a whole (both 16%) [1]. Every year, more than 22500 of its people are killed by tobacco-caused diseases. Still, more than 16000 children aged 10–14 years and nearly 2.5 million adults (15+ years old) continue to use tobacco each day [2]. Cigarette smoking also causes a reduced health-related quality of life (QoL). Smoking abstinence significantly improves the health-related quality of life among tobacco users before and after smoking cessation [3].

To tackle this public health problem, Kazakhstan ratified the Framework Convention on Tobacco Control in 2006. Despite these efforts, tobacco-related premature deaths in Kazakhstan increased to >50% of all smokers in 2014 [4]. The World Health Organization (WHO) and its member states have set a voluntary global target of a 30% relative reduction in the prevalence of current tobacco use by 2025 [5]. One of the MPOWER strategies of tobacco control is to offer help to quit tobacco dependence. This effort utilizes different strategies, including both non-pharmacological (behavioural counselling) and pharmacological therapies. Currently, it is well-accepted that smoking cessation drugs such as Nicotine Replacement Therapy (NRT) are effective and safe in real-world settings [6]. When a person stops using tobacco, the aim of NRT is to replace the nicotine that the smoker would have been receiving, without the additional harmful elements of tobacco [7]. With fast-acting drugs such as nicotine gum, the nicotine is absorbed through buccal mucosa and reaches peak plasma nicotine concentrations within 15–30 min (as compared to within 1–2 min after smoking) and the blood levels reach a flat peak after about 30 minutes of chewing [8]. On the contrary, trans-dermal patches release nicotine more gradually with peak concentrations within hours after application [9]. As a result, none of the available NRT products delivers such high doses of nicotine as quickly as cigarettes. One implication is that combination NRT (fast-acting form + patch) results in approximately 15% to 36% higher long-term quit rates than a single form of NRT [7].

Using a nicotine replacement treatment (or other smoking cessation drugs) before a quit attempt while smoking normally is called preloading. A systematic review showed limited evidence that nicotine preloading may be effective, but with unexplained heterogeneity between studies. Some studies suggested that nicotine preloading doubled the likelihood of achieving abstinence, while other studies suggested that preloading had no effect [10]. However, more evidence is needed to determine the effectiveness of preloading combination NRT when compared with the standard regimen. Hence, this study aims to determine the effectiveness of preloaded combination NRT on smoking cessation of tobacco smokers, the change in health-related quality of life due to smoking cessation and to explore the perception on treatment adherence.

## Materials and methods

### Study design

**Fig 1:**
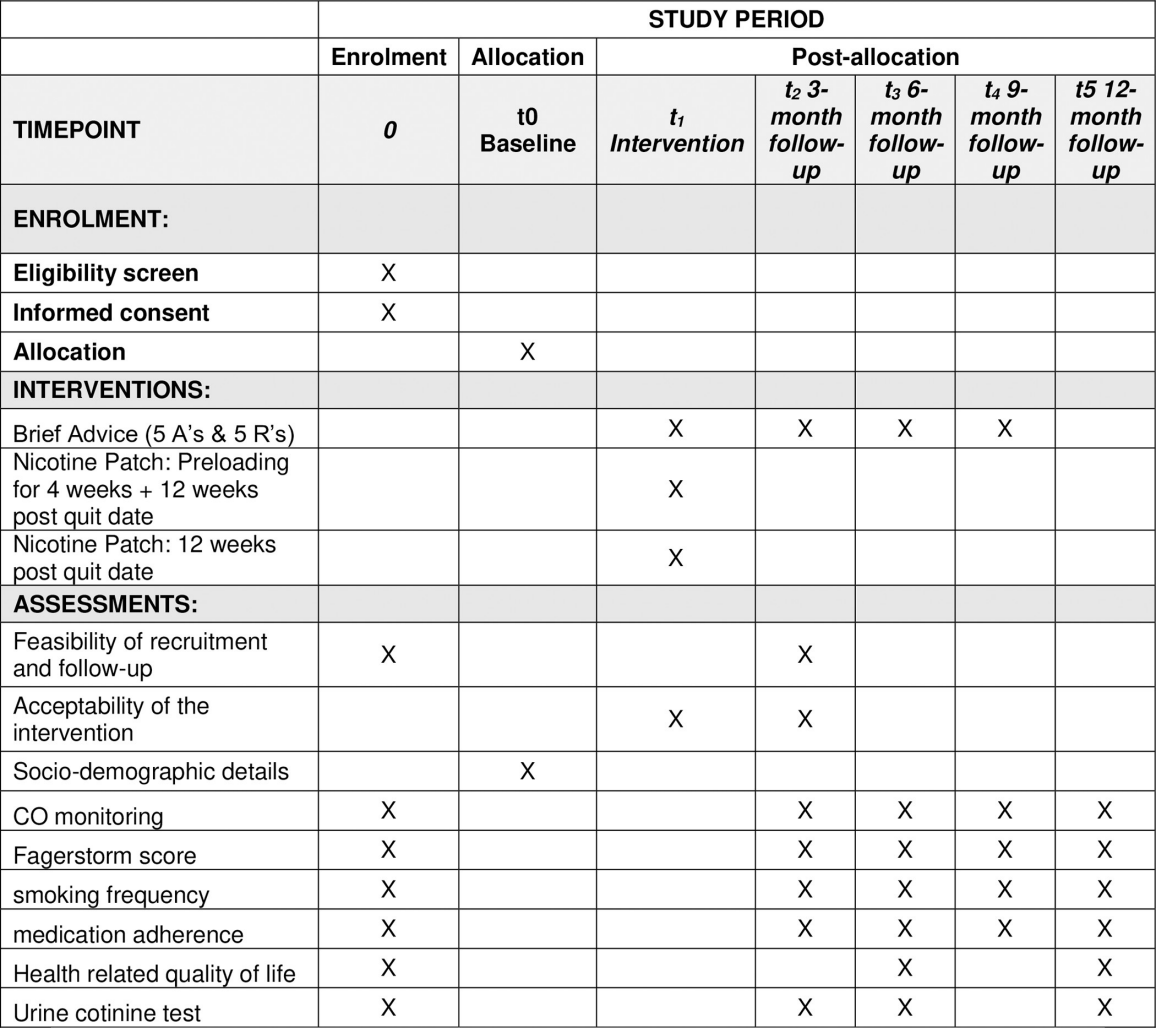
Content for the schedule of enrolment, interventions, and assessments.

This randomized, single-blind study protocol was designed based on Consolidated Standards of Reporting Trials (CONSORT) 2010 Guidelines. This superiority trial intends to gather further evidence that combination nicotine replacement therapy is more efficient than a fast-acting NRT alone. A SPIRIT schedule of enrollment, interventions, and assessments is shown in **Fig 1**.

### Study setting

The study will be conducted at the National Research Cardiac Surgery Center, Astana, Kazakhstan. This hospital is a tertiary-level facility with more than 700 beds. As part of developing the infrastructure for the study, an exclusive tobacco cessation centre will be established in a carpet area of 500–600 sq. ft in the hospital premises.

### Study population

All patients and/or visitors aged 18 years and above at the National Research Cardiac Surgery Center in Astana, Kazakhstan, will undergo screening for tobacco use. For the purpose of this study, smokers will be defined as individuals who have been smoking more than 10 cigarettes per day for six months or less, and they must have a motivation to quit. We will include all willing smokers in the study.

### Sample size calculation

In a study conducted to find the efficacy of preloading combination NRT over standard combination NRT, 6-month sustained abstinence was 47.5% for the intervention group and 27.5% for the control group (effect size of 20%) with a level of significance of 5%, with a power of 80% in the two-sided test [11]. This proportion was considered to calculate the sample size for the current study. Eventually, the sample size was derived at 91 in each arm. This sample size was calculated using the sample size formula for the clinical trial studies described in Kelsey et al. [12]. We have used OpenEpi version 3.01 to calculate the sample size [13]. After considering a loss to follow-up of 10% in each arm, the final sample size for the study per arm is 100.

### Randomization, blinding, and allocation concealment

Block randomization will be done to obtain the two groups using a computer-generated table. Each block contains four participants in a unique sequence of experimental arm and control arm and the total number of blocks will be 50. The participant and the principal investigator will not be aware of the group allocation. Data will be maintained by the senior research fellow. Concealment will be ensured in each participant’s group allocation by a sealed envelope with the identity number labelled on the envelope.

### Study procedure

**Fig 2:**
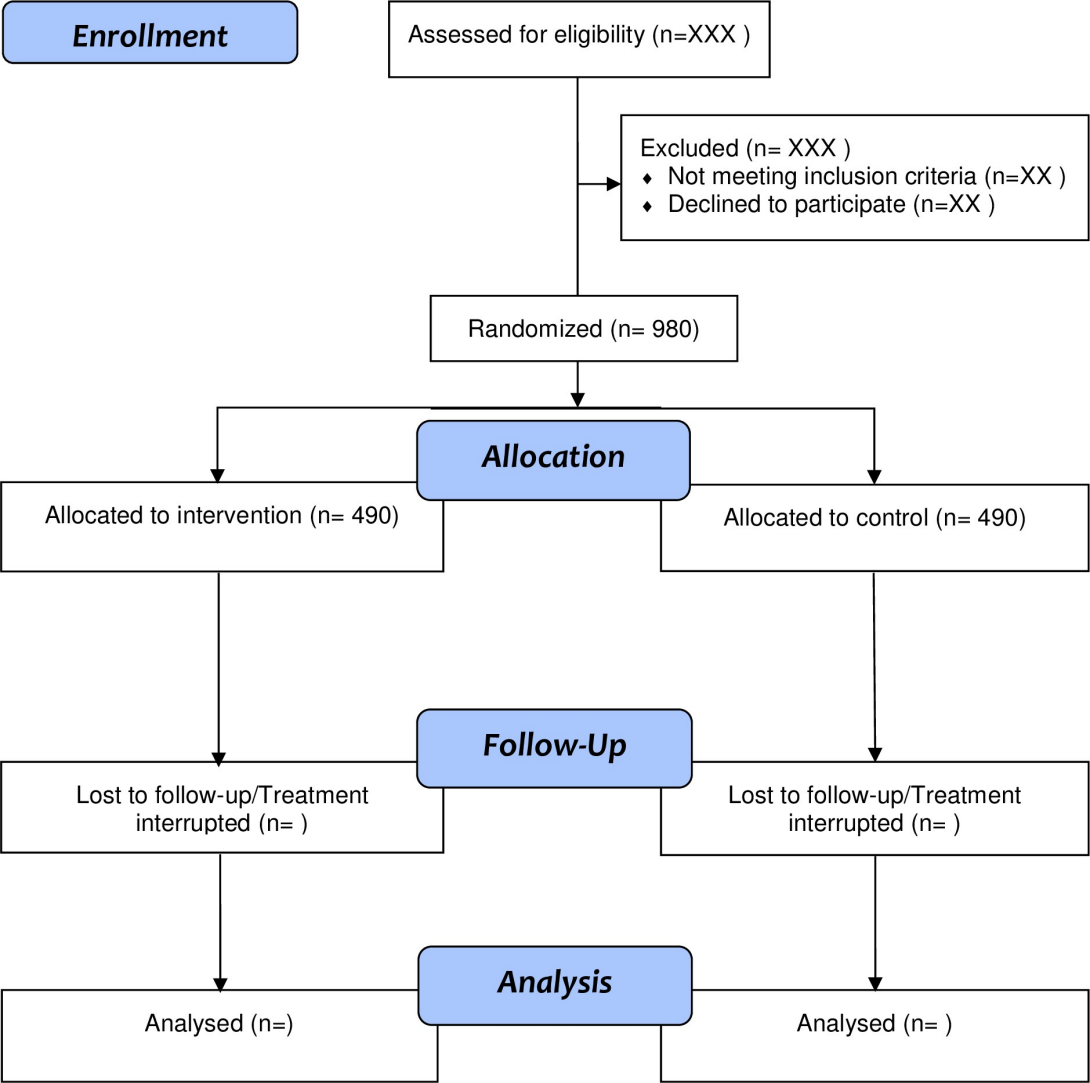
Flow diagram depicting the selection of participants to preloading combination nicotine replacement therapy trial.

The section of participants is depicted in **Fig 2**. Those who are known tobacco smokers will be advised to register for cessation services at the tobacco cessation centre. The smoking cessation trial aims to recruit 490 participants in each arm. The participants will be enrolled based on their eligibility (all current smokers aged 18 years and above with a motivation to quit). Before randomization, smokeless tobacco users, smoking frequency of fewer than 10 cigarettes per day, pregnant women, lactating women, patients with a recent history of myocardial infarction of fewer than 3 months, and electronic cigarette users will be excluded from the study. Further to this, randomization will be done to allocate participants to the appropriate study arms.

### Intervention

A full-time psychologist shall provide the counselling services and a qualified medical practitioner shall prescribe the nicotine replacement therapy. It is comparable to receiving treatment with varenicline when slow-release NRT (the patch) is combined with rapid-release NRT (nicotine gum, lozenge, inhaler, or nasal spray). The use of nicotine replacement therapy while smoking or in combination with other treatments is regarded as safe. The intervention arm is the combination nicotine replacement therapy. This includes a fixed dose combination of nicotine patch and 4 mg nicotine chewing gum for 12 weeks on a tapering dosage pattern. The control arm includes 4 mg nicotine chewing gums alone for 12 weeks on a tapering dosage pattern.

Based on the WHO-5As model of brief advice, group counselling (Ask, Advise, Assess, Assist, Arrange) will be arranged for the participants in both arms to quit in a clear, strong and personalized manner. Then, smokers will be assessed for their readiness of quitting. For those willing to quit, the study team would assist the participants with the interventional quitting plans as per the allocated arm. The frequency of follow-up for behavioral support will be arranged every two weeks. Supplementary cessation assistance such as social media and self-help materials will be provided. For those not willing to quit, 5Rs (Relevance, Risks, Rewards, Roadblocks, Repetition) motivational counselling intervention will be provided at least three times with a gap of one week in every group counselling until the participants were ready to quit [14].

Carbon monoxide (CO) monitor will be used to assess the exhaled CO level of the study participants during each visit. The cutoff point will be six parts per million or less for nonsmokers and more than six parts per million for smokers [15]. Cotinine is a metabolite of nicotine with a long half-life (15–19 hours) that can be measured in body fluids such as saliva, urine and plasma. Measurement of salivary cotinine as an indicator of tobacco use, exposure to secondhand smoke or use of pharmaceutical nicotine is commonly used in population studies, given the ease of sample collection and minimal discomfort to study subjects [16]. Hence, saliva cotinine test will be done to assess the smoking status of the participants during baseline and end-line of the study. Saliva cotinine test results will be considered as the biochemical verification for smoking status. However, carbon monoxide levels will be a complementary test to support the evidence for the smoking status of the participants. This is because the concentration of cotinine measured in plasma, saliva, or urine has the highest sensitivity and specificity to detect smokers in comparison to CO concentration in blood and expired air due to relatively lower sensitivity than cotinine [17]. The detailed description of the intervention is shows in **Table 1**.

**Table 1:** Therapy protocol for the experimental and control groups.

Activity	Experimental Group	Control Group
Brief Advice (5 A’s & 5 R’s)	0-day, Month- 1, 3, 6, 9, 12	0-day, Month- 1, 3, 6, 9, 12
Nicotine Replacement Therapy for > 20 cigarettes per day	Nicotine Patch: Preloading for 4 weeks + 12 weeks post quit date **21 mg: 4 weeks before and after quit date** • 14 mg: Next 4 weeks • 7 mg: Last 4 weeks Nicotine chewing gums 4 mg for 12 weeks post quit date (12/day on week 1………….1/day on week 12	Nicotine Patch: 12 weeks post quit date • 21 mg: 4 weeks after quit date • 14 mg: Next 4 weeks • 7 mg: Last 4 weeks Nicotine chewing gums 4 mg for 12 weeks post quit date (12/day on week 1………….1/day on week 12
**Nicotine Replacement Therapy for < 20 cigarettes per day**	Nicotine Patch: Preloading for 4 weeks + 12 weeks post quit date • 21 mg: 4 weeks before and after quit date • 14 mg: Next 4 weeks • 7 mg: Last 4 weeks Nicotine chewing gums 2 mg for 12 weeks post quit date (12/day on week 1………….1/day on week 12	Nicotine Patch: 12 weeks post quit date • 21 mg: 4 weeks after quit date • 14 mg: Next 4 weeks • 7 mg: Last 4 weeks Nicotine chewing gums 2 mg for 12 weeks post quit date (12/day on week 1………….1/day on week 12
CO monitoring, Fagerstorm score, smoking frequency, medication adherence	0-day, Month- 1, 3, 6, 9, 12	0-day, Month- 1, 3, 6, 9, 12
Health related quality of life	0-Day, 6 months, 12 months	0-Day, 6 months, 12 months
Urine cotinine test	0-day, Month- 3, 6, 12	0-day, Month- 3, 6, 12

### Study tools

A semi-structured questionnaire will be developed to capture information about the participants’ socio-demographic factors, smoking history, current smoking status, tobacco dependence score, medical history, follow-up of treatment, and stage of the transtheoretical model during each visit. The participants will be assessed for carbon monoxide levels, body weight in kg during each visit and salivary cotinine test during the start and end of the follow-up. Processes of change questionnaire, Fagerstrom test questionnaire, medication adherence rating scale, and clinician rating scale will be used in the study. Predictors of smoking cessation such as concern about own health, others’ health due to passive smoking, smoking expenses, past quit attempts, etc will be measured.

### Outcome variables

The primary clinical outcome will be smoking cessation (abstinence or non-abstinence) assessed at 12 months from the starting point of intervention. Non-abstinence will be defined as self-reported smoking anytime during the intervention or follow-up and a positive salivary cotinine test.

Secondary outcomes of interest at the final follow-up will include the number of quit attempts since enrollment, the number of smoked cigarettes/bidis per day (for those who were not able to quit), and the duration of use of the intervention. The tobacco use outcomes will be smoking excess, smoking reduction, continued use and relapse from the baseline information. Interim smoking cessation outcomes will be assessed at 3 and 6 months of intervention.

### Statistical analysis

Paper-based data collection will be entered into Microsoft Excel sheets, and STATA version 14/R software will be used for analysis. For qualitative data, frequency and percentage will be used to summarise the data, and for quantitative data, mean and standard deviation. A paired t-test will be used to compare the quantitative outcome measures before and after the intervention. The Wilcoxon Signed Rank test will be used if the distribution of the data does not fit that of normality. The Chi-square test or Fischer exact test will be used to compare qualitative results. A P value less than 0.05 will be considered as statistically significant.

To address missing data in our study, we will employ multiple imputation techniques. We will use response imputation for the outcome variable and covariate missingness imputations for the explanatory variables. Sensitivity analyses will be conducted to evaluate the impact of missing data on the study outcomes. For handling longitudinal dichotomous data with substantial missing data, specifically when missing data constitutes more than 10% of the total sample size, we will consider the use of a Pattern-Mixture Model within a mixed-effects logistic regression framework. The Pattern-Mixture Model allows us to account for different patterns of missingness and model the potential impact of missing data on the study results. The mixed-effects logistic regression model will be utilized for analyzing the dichotomous outcome since it effectively accommodates the repeated measures nature of the data and the binary nature of the outcome variable.

We will conduct a survival analysis to assess the variation in smoking relapse over time, considering socio-demographics, smoking history, and treatment-related variables. We will use both log-rank tests and Cox proportional hazards models to analyze the data. In case of violations of the Cox proportional hazards assumptions, we will explore alternative models such as the accelerated failure time model or Cox models with time-varying coefficients. Time-to-event will be defined as the duration from the start of the smoking cessation program to the occurrence of smoking relapse. Censoring will occur when participants do not experience a relapse during the study period or are lost to follow-up. We will handle censoring appropriately during the analysis, using the Kaplan-Meier estimator to estimate survival probabilities and accounting for censored data in the Cox proportional hazards models.

### Ethical considerations

The study protocol was reviewed and approved by the Institutional Central Ethics Committee of the University and the approval number is 495/23112021. The study was registered with the Clinical Trials Registry. Administrative approval will be obtained from the hospital authorities. Participation in the study will be voluntary and written informed consent will be obtained by the study participants. Participants are free to withdraw from the study at any point in time. All the participants will be given brief advice. The Control group will be given standard treatment after completion of the trial. Side effects if any will be reported to the ethics committee and arranged for management at the Hospital. The cost of treatment will be borne by the investigator. The study has no conflict of interest during any stage of the trial.

### Safety considerations

We do not anticipate any adverse events (AEs) for this trial. However, the safety aspects of the study are closely monitored by the Data and Safety Monitoring Board (DSMB), which receives unblinded data for its judgment. In the event of any serious adverse event (SAE), it will be promptly reported.

### Patient and public involvement

Patients and/or visitors of the hospital will be screened for tobacco use. Those who are known tobacco smokers will be advised to register for cessation services at the tobacco cessation centre.

Participation in the study will be entirely voluntary, and all study participants will be required to provide written informed consent. Before obtaining consent, the study objectives and purpose will be thoroughly explained to the potential participants. It is important to note that patients were not involved in the design of this study. The recruitment of patients will take place at the National Research Cardiac Surgery Center, located in Astana, Kazakhstan. This esteemed center will serve as the primary location for gathering participants for the study.

## Discussion

This study focuses on determining the effectiveness of preloaded combination nicotine replacement therapy on smoking cessation in the adult population. The study results shall add scientific evidence to the available literature about whether the preloaded combination of nicotine replacement therapy is effective. This study can potentially provide valuable insights for future researchers in the same area of interest. They may choose to adopt certain or most aspects of the study protocol that are relevant to their own methodology and research setup. By drawing from the findings and procedures of this study, future researchers can enhance the robustness and applicability of their investigations. This will contribute to advancing knowledge and understanding in the field, fostering further progress in research and patient care. The study focuses on establishing high certainty evidence about the effectiveness of preloading combination nicotine replacement therapy on smoking cessation in the adult population. With this outcome, the study shall help the medical practice to improve the cessation rates among tobacco smokers thereby reducing the costs incurred by the health systems. Future systematic reviews and meta-analyses shall develop high certainty evidence from this study which in turn helps the medical professionals to benefit the smoker’s community through effective cessation prescriptions.

### Strengths and limitations of this study

- The study focuses on establishing high certainty evidence about the effectiveness of preloading combination nicotine replacement therapy on smoking cessation of the adult population; with this outcome, the study shall help the medical practice to improve the cessation rates among tobacco smokers thereby reducing the costs incurred by the health systems.
- The expected results of this project will improve our understanding of the Afterload of nicotine replacement therapy and will have a significant contribution to further developing the study in the Adult population of Kazakhstan.
- As there is no universally accepted therapy regimen for nicotine replacement therapy, the effectiveness of nicotine replacement therapy plays a crucial role in improving the quit rates among smokers which may be a primary level of prevention of tobacco-related diseases.
- Since this research is conducted in the community using novel therapy regimens, the lack of blinding of the assessors collecting data is a limitation to the study design.

## Supporting information

S1 Checklist (DOC)

S1 File (PDF)
