# Supplementary material for: Preloaded combination nicotine replacement therapy for smoking cessation in Kazakhstan: A randomized controlled trial study protocol
Source: PLoS One. 2023 Nov 27;18(11):e0292490. doi: 10.1371/journal.pone.0292490 (PMC10681200; doi:10.1371/journal.pone.0292490)
Supplement: S1 File — (PDF) [file pone.0292490.s002.pdf]

RESEARCH PROPOSAL FORM*

Project Information

Does this project involve:	**Yes** ☐	**No** ☐
(1) Human Subjects	☒	☐
(2) Animal Subjects	☐	☐
(3) Radiological Materials	☐	☐
(4) Hazardous Materials	☐	☐
(5) Increased Need for Space/Utilities	☒	☐
(6) Commercial Organization/Foundation	☐	☐
(7) Intellectual property	☐	☐
(8) PI had Social policy grant	☐	☐

**1. General information**	
Project	
Title:	**Effectiveness of preloaded combination nicotine replacement therapy on smoking cessation of adult population in Kazakhstan- A randomized controlled trial**
Proposed start and final date:	January 2022 to December 2024
Duration:	3 years
Location:	Nur-Sultan, Kazakhstan
Requested amount of funding, in USD:	421684
Principal Investigator	
Name:	Chethan Purushothama

* Note that NU requirement for the external peer review purposes: Research Proposal (including tables, charts and bibliography) should not exceed 15 pages limit. Please submit complete proposals in PDF format. Proposal supporting data consists of a 2 page CV (also in PDF) of the Principal Investigator and possibly other 1 page CV's of other key personnel.

Faculty:	School of Sciences & humanities

2. Project description

Background:

Tobacco use is the world's leading preventable cause of premature death and disease causing over 5 million deaths annually around the globe. The proportion of deaths due to smoking in Kazakhstan in 2004 was 24%, twice the worldwide rate (12.0%) and about 50% higher than the rates in Eurasia and in the Russian Federation as a whole (both 16%) **[1].** Tobacco harms the health, the treasury, and the spirit of Kazakhstan. Every year, more than 22500 of its people are killed by tobacco-caused disease. Still, more than 16000 children aged 10-14 years and nearly 2.5 million adults (15+ years old) continue to use tobacco each day **[2].** Cigarette smoking also causes reduced health-related quality of life (QoL). Smoking abstinence significantly improves the health-related quality of life among tobacco users before and after smoking cessation (health transition) **[3].**
To tackle this public health problem, Kazakhstan ratified the Framework Convention on Tobacco Control in 2006. In spite of these efforts, the projected tobacco-related premature deaths in Kazakhstan increased to >50% of all smokers in 2014 **[4].** The World Health Organization (WHO) and its member states have set a voluntary global target of a 30% relative reduction in the prevalence of current tobacco use by 2025. This organization provides technical guidance based on six demand-reduction measures of MPOWER such as- monitor tobacco use and prevention policies; protect people from tobacco smoke; offer help to quit tobacco use; warn about the dangers of tobacco; enforce bans on tobacco advertising, promotion and sponsorship; and raise taxes on tobacco. **[5]**

One of the MPOWER strategies is to offer help to quit tobacco dependence. This effort utilizes different strategies including both non-pharmacological (behavioral counselling) and pharmacological therapies. Currently, it is well accepted that smoking cessation drugs such as Nicotine Replacement Therapy (NRT) are effective and safe in real-world settings. **[6]**

When a person stops using tobacco, the aim of NRT is to replace the nicotine that the smoker would have been receiving, without the additional harmful elements of tobacco.**[7].** With fast acting drugs such as nicotine gum, the nicotine is absorbed through buccal mucosa and reaches peak plasma nicotine concentrations within 15–30 min (as compared to within 1–2 min after smoking) and the blood levels reach a flat peak after about 30 minutes of chewing **[8].** On the contrary, trans-dermal patches release nicotine more gradually with peak concentrations within hours after application **[9].** As a result, none of the available NRT products deliver such high doses of nicotine as quickly as cigarettes. One implication is that combination NRT (fast-acting form + patch) results in approximately 15% to 36% higher long-term quit rates than a single form of NRT. **[7]**

Using a nicotine replacement treatment (or other smoking cessation drugs) before a quit attempt while smoking normally is called preloading. A systematic review showed limited evidence that nicotine preloading may be effective, but with unexplained heterogeneity between studies. Some studies suggested that nicotine preloading doubled the likelihood of achieving abstinence, while other studies suggested that preloading had no effect **[10].** However, more evidence is needed to determine the effectiveness of preloading combination NRT when compared with standard regimen **[7].** This study intends to determine and gather further evidence about the effectiveness of preloading combination NRT among adult smokers of Kazakhstan.

Research question: What is the effectiveness of preloaded combination nicotine replacement therapy on smoking cessation?

3. Methodology and ethical considerations

Objectives:

- To determine the effectiveness of preloaded combination nicotine replacement therapy (NRT) on smoking cessation of tobacco smokers.
- To determine the predictors of smoking cessation among the study participants.
- To determine the change in health-related quality of life due to smoking cessation.
- To determine the perception of treatment adherence among the study participants.

Methodology:

A. **Trial Design:** This randomized, controlled, two-armed, single-blinded, superiority trial with 1:1 allocation ratio is designed based on CONSORT 2010 Guidelines. This superiority trial intends to gather further evidence that combination nicotine replacement therapy is more efficient than a fast-acting NRT alone.

B. **Study Setting:**

The study will be conducted at the National research cardiac surgery Centre, Turan avenue, Nur-Sultan, Kazakhstan. This hospital is a tertiary level facility with more than 200 beds. As part of developing the infrastructure for the study, an exclusive tobacco cessation center will be established in a carpet area of 500-600 square feet in the hospital premises. A full-time psychologist shall provide the counseling services and a qualified medical practitioner shall prescribe the nicotine replacement therapy.

C. **Recruitment of participants:**

Patients and/or visitors of the hospital will be screened for tobacco use. Those who are known tobacco smokers will be advised to register for cessation services at the tobacco cessation center. The smoking cessation trial aims to recruit 490 participants in each arm. The participants will be enrolled based on the eligibility (all current smokers aged 18 years and above with a motivation to quit). Before randomization, smokeless tobacco users, smoking frequency of less than 10 cigarettes per day, pregnant women, lactating women, patients with recent history of myocardial infarction of less than 3 months, and electronic cigarette users will be excluded from the study. Further to this, randomization will be done to allocate participants to the appropriate study arms. The participant information sheet will be given and explained to the participants by the investigator about the risks and benefits of participating in the trial.

D. Intervention and Control arm:

Combining slow-release NRT (the patch) with rapid-release forms (nicotine gum, lozenge, inhaler, or nasal spray) increases the effectiveness of NRT and is comparable with treatment with varenicline. Nicotine replacement therapy is considered safe, including in combination form or if used while smoking. Among the different forms of NRT, adherence is greatest with the patch.**[11].** The intervention arm is the combination nicotine replacement therapy. This includes a fixed dose combination of nicotine patch and 4 mg nicotine chewing gum for 12 weeks on tapering dosage pattern. The control arm includes 4 mg nicotine chewing gums alone for 12 weeks on tapering dosage pattern.

Based on the WHO-5As model of brief advice, group counseling (Ask, Advise, Assess, Assist, Arrange) will be arranged for the participants in both the arms to quit in a clear, strong and personalized manner.Then, smokers will be assessed for the readiness of quitting. For those willing to quit, the study team would assist the participants with the interventional quitting plans as per the allocated arm. The frequency of follow up for behavioral support will be arranged every two weeks. Supplementary cessation assistance such as social media and self-help materials will be provided. For those not willing to quit, 5Rs (Relevance, Risks, Rewards, Roadblocks, Repetition) motivational counseling intervention will be provided at least three times with a gap of one week in every group counseling until the participants were ready to quit **[12.]**

Carbon monoxide (CO) monitor will be used to assess the exhaled CO level of the study participants during each visit. The cutoff point will be six parts per million or less for nonsmokers and more than six parts per million for smokers [15]. Cotinine is a metabolite of nicotine with a long half-life (15–19 hours) that can be measured in body fluids such as saliva, urine and plasma. Measurement of salivary cotinine as an indicator of tobacco use, exposure to secondhand smoke or use of pharmaceutical nicotine is commonly used in population studies, given the ease of sample collection and minimal discomfort to study subjects.**[13].** Hence, saliva cotinine test will be done to assess the smoking status of the participants during baseline and end-line of the study. Saliva cotinine test results will be considered as the biochemical verification for smoking status. However, carbon monoxide levels will be a complementary test to support the evidence for smoking status of the participants. This is because the concentration of cotinine measured in plasma, saliva, or urine has the highest sensitivity and specificity to detect smokers in comparison to CO concentration in blood and expired air due to relatively lower sensitivity than cotinine **[14]**

E. **Primary Outcomes:**

The primary clinical outcome will be the binary smoking cessation; abstinence or non-abstinence assessed at 12 months from the starting point of intervention. Non-abstinence will be defined as self-reported smoking anytime during the intervention or follow up and a positive salivary cotinine test.

F. Secondary outcomes:

Secondary outcomes of interest at the final follow-up will include number of quit attempts since enrollment, the number of smoked cigarettes/bidis per day (for those who were not able to quit), duration of use of intervention. The tobacco use outcomes will be smoking excess, smoking reduction, continued use and relapse from the baseline information. Interim smoking cessation outcomes at 3 and 6 months of intervention will be assessed.

G. Sample size:

In a study conducted to find the efficacy of preloading combination NRT over standard combination NRT, 6-month sustained abstinence was 47.5% for the intervention group and 27.5% for the control group with a level of significance of 5%, power of 80% in the two-sided test **[15.].** This proportion was considered to calculate the sample size for the current study. Eventually, the sample size was derived at 91 in each arm. By considering a loss to follow up of 10% in each arm, the sample size is 100.1 and hence rounded off conveniently to 100 per arm.

H**. Randomization, blinding, and allocation concealment:**

When planning a randomized clinical trial, careful caution must be executed in the selection of participants for various arms of the study. Selection and accidental bias may occur when participants are not assigned to study groups with equal probability. Hence, block randomization will be done to obtain the two groups using a computer-generated table. Each block contains four participants in a unique sequence of experimental arm and control arm of 245 blocks. The trial will be single-blinded wherein the participant and the principal investigator will not be aware of the group allocation. Data will be maintained by the senior research fellow. Concealment will be ensured ineach participant's group allocation by a sealed envelope with the identity number labeled on the envelope.

I. Study tools:

A semi-structured questionnaire will be developed to capture information about the participants' socio-demographic factors, smoking history, current smoking status, tobacco dependence score, medical history, follow-up of treatment, and stage of transtheoretical model during each visit. The participants will be assessed for carbon monoxide levels, body weight in kg during each visit and salivary cotinine test during the start and end of the follow-up. A pilot study will be conducted on ten participants to test the validity and feasibility of the tools. Processes of change questionnaire, fagerstorm test questionnaire, medication adherence rating scale, and clinician rating scale will be used in the study. Predictors of smoking cessation such as concern about own health, others' health due to passive smoking, smoking expenses, past quit attempts, etc will be measured. **[16].**

J. Therapy protocol:

Activity	Experimental Group	Control Group
Brief Advice (5 A's & 5 R's)	0-day, Month- 1, 3, 6, 9, 12	0-day, Month- 1, 3, 6, 9, 12
NicotineReplacement Therapy for > 20 cigarettes per day	Nicotine Patch: Preloading for 4 weeks + 12 weeks post quit date<br>- 21 mg: 4 weeks before and after quit date<br>- 14 mg: Next 4 weeks<br>- 7 mg: Last 4 weeks<br><br>Nicotine chewing gums 4 mg for 12 weeks post quit date (12/day on week 1…………1/day on week 12	Nicotine Patch: 12 weeks post quit date<br>- 21 mg: 4 weeks after quit date<br>- 14 mg: Next 4 weeks<br>- 7 mg: Last 4 weeks<br><br>Nicotine chewing gums 4 mg for 12 weeks post quit date (12/day on week 1…………1/day on week 12
Nicotine Replacement Therapy for < 20 cigarettes per day	Nicotine Patch: Preloading for 4 weeks + 12 weeks post quit date<br>- 21 mg: 4 weeks before and after quit date<br>- 14 mg: Next 4 weeks<br>- 7 mg: Last 4 weeks<br><br>Nicotine chewing gums 2 mg for 12 weeks post quit date (12/day on week 1…………1/day on week 12	Nicotine Patch: 12 weeks post quit date<br>- 21 mg: 4 weeks after quit date<br>- 14 mg: Next 4 weeks<br>- 7 mg: Last 4 weeks<br><br>Nicotine chewing gums 2 mg for 12 weeks post quit date (12/day on week 1…………1/day on week 12
CO monitoring, Fagerstorm score, smoking frequency, medication adherence	0-day, Month- 1, 3, 6, 9, 12	0-day, Month- 1, 3, 6, 9, 12
Health related quality of life	0-Day, 6 months, 12 months	0-Day, 6 months, 12 months
Saliva cotinine test	0-day, Month- 3, 6, 12	0-day, Month- 3, 6, 12

K.Statistical Analysis: The collected information will be summarized by using frequency and percentage for qualitative data, mean and standard Deviation for quantitative data. To compare the outcome measures, quantitative before and after intervention, paired-t test will be used. If data is not following normal distribution, Wilcoxan Signed Rank test will be used. To compare qualitative outcomes, Chi-square test or Fischer exact test will be used.To compare the difference in outcome measures between two intervention groups, independent sample‘t’ test will be used. If data is not following normal distribution, Mann Whitney test will be used. The P value less than 0.05 will be considered as statistically significant.

Results: The study shall adopt the "Intention to Treat Analysis" mechanism. If the study has missing data more than 10% of the total sample size as expected during sample size calculation, Pattern-Mixture Model within a mixed-effects logistic regression model for longitudinal dichotomous data will be used. Because of the dichotomous outcome, logistic regression version of the mixed-effects model is applicable. The pattern-mixture effects for drug, time, data pattern and their interactions will be interpreted. Pattern mixture sensitivity analysis using SAS/STAT, missing data analysis will be done using R software, and survival analysis will be used to analyze the variation in smoking relapse over time related to the socio-demographics, smoking history, and treatment-related variables.

Declarations:

Ethical approval and consent to participate

Approval will be obtained from the Institutional Central Ethics Committee of the University. The study will be registered with the Clinical Trials Registry. Administrative approval will be obtained from the hospital authorities. Participation in the study will be voluntary and written informed consent will be obtained by the study participants. Participants are free to withdraw from the study at any point of time. All the participants will be given brief advice. Control group will be given standard treatment after completion of the trial. Side effects if any will be reported to the ethics committee and arranged for management at the Hospital. The cost of treatment will be borne by the investigator. The study has no conflict of interest during any stage of the trial.

The trial investigators will update the progress on a regular basis to take necessary measures to avoid deviations and/or to take approvals in case of any deviations required. Adverse events and serious adverse events will be reported to the clinical committee and the ethics committee. Data management will be monitored by the principal investigator to check for any falsification or fabrication of results. Plagiarism software will be used for writing scientific articles. Any change in the authorship will be intimated to the scientific committee for necessary approvals.

Conclusion:

This study focuses on determining the effectiveness of preloaded combination nicotine replacement therapy on smoking cessation of adult population. The study results shall add scientific evidence to the available literature about the preloaded combination nicotine replacement therapy being an effective. The study protocol through publication in leading journals assists future researchers in this area of interest to adopt certain or most of the aspects of the study protocol relevant to their methodology and research setup.

Flow diagram: Preloading combination Nicotine Replacement Therapy trial

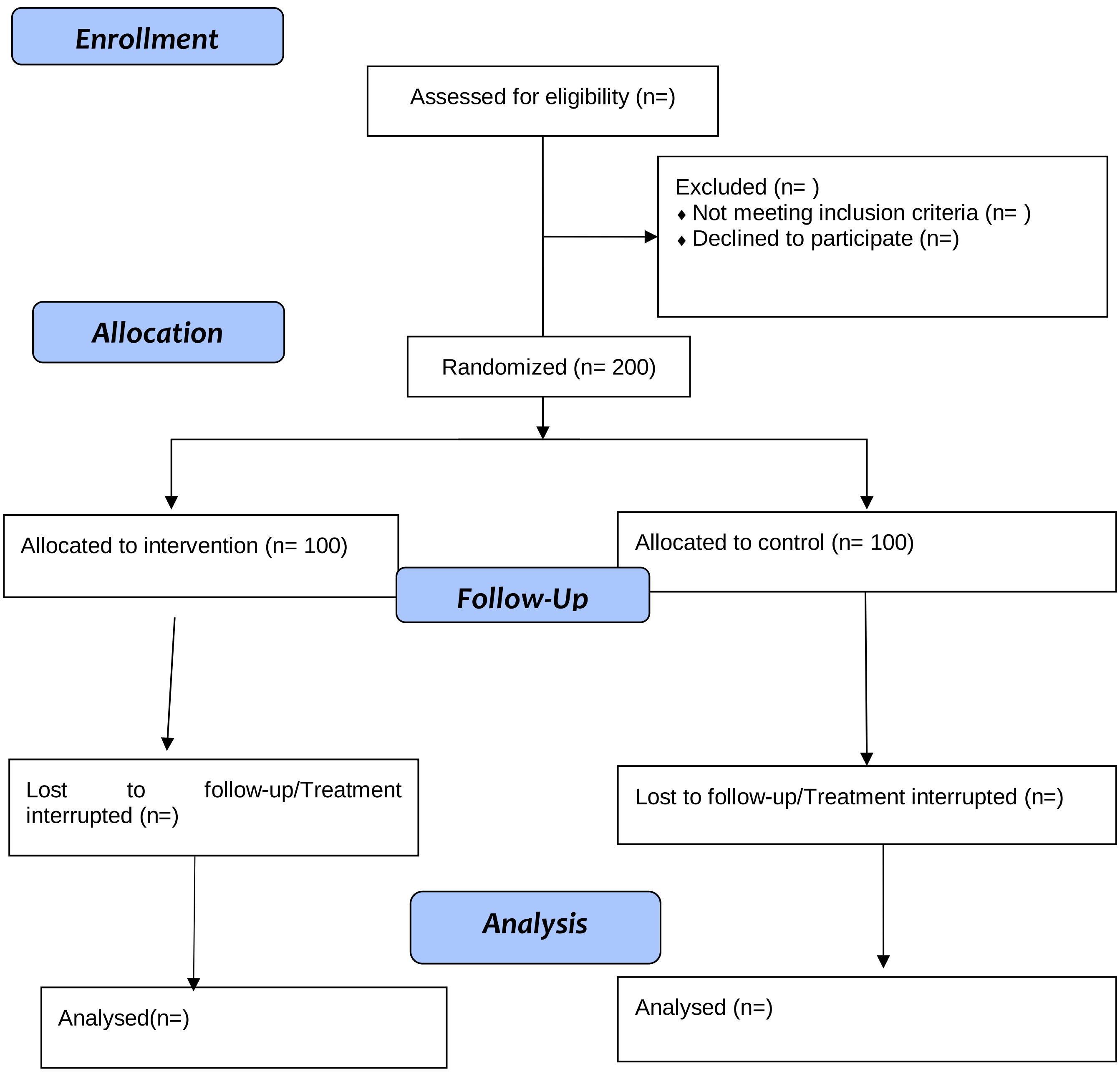

4. Project plan and budget justification

The Principal Investigator (PI) shall be the technical, administrative, and financial decision maker for the research project. The co-PI shall work in partnership with the PI and the other team members such as master's degree holders and students shall assist both the PI and the co-PI. The PI shall be the competent authority to report to the organizations about the progress, modifications, adverse reactions, or any other issues that arise during the course of the project.

The project duration will span across 3 years starting January 2022. The project will have a continuous schedule throughout the year similarly to the working hours of the project site. Proposed time table & events for 3 years are as follows:
The Infrastructure for the study will be setup in 60 days from January 2022 to April 2022. A room size of 500/600 square feet will be identified at the cardiac research center for counselling & treatment for the participants.
Recruitment of participants will be done between April 2022 and June 2023. Potential participants will be patients attending the OPD and those discharged from the IPDs of the health facility. They will be screened for tobacco use based on a brief questionnaire method. Those eligible for the study will be explained about the study purpose to convert the eligibility to participation.

The trial begins from April 2022 and is proposed to end in June 2024 including the follow up of the last recruited participant. The duration of the trial including follow up is 12 months.

TITLE	START	FINISH	DURATION
1.Infrastructure setup	2022-01-03	2022-04-02	90 days
2.Procurement of equipment/drugs	2022-02-01	2022-04-01	60 days
3.Recruitment of human resources	2022-01-17	2022-04-01	75 days
4.Sensitization Workshop	2022-04-01	2022-04-01	1 days
5.Recruitment of participants	2022-04-01	2023-06-24	450 days
6.Review meeting	2022-06-01	2022-06-01	1 days
7.Conduct of the trial including follow up	2022-04-01	2024-06-23	815 days
8.Review meeting	2022-11-01	2022-11-01	1 days
9.Interim data analysis and publications	2023-07-03	2023-12-29	180 days
10.Review meeting	2023-07-20	2023-07-20	1 days
11.Final data analysis	2024-06-24	2024-08-22	60 days
12.Report writing	2024-08-23	2024-10-21	60 days
13.Report submission and data dissemination	2024-10-22	2024-12-20	60 days
14.Project Closure	2024-12-31	2024-12-31	1 days

Budget justification

As shown in details in Appendix C, the total project cost for three years is categorized as follows.

ITEM	FUND (USD)
Payment to research team	109200
Travel	14400
Materials and supplies	228400
Equipment	14600
Publication costs, registration, dissemination costs	25000
Subcontracts	9000
Overheads (5%)	21084
Total	421684

The justification of each item are as follows:

1) Payment to the research team: Our research team consists of MPH graduates/students from NUSOM, Nazarbayev university. Students & graduates of Nazarbayev university will enhance their knowledge in the field of tobacco control & also, they will research publish papers in this area. The payment at this section will be allocated to students /Graduates during their research.

2) Travel: The travel will cover conference expenses of the Investigators.

3) Equipment: The equipment needed to buy for the project is as follows: Portable spirometers, carbon monoxide monitors, sphygmomanometers, and weighing scale, all two each in numbers will be procured. To stablish an office setup, furniture & computers will be procured. All the equipment mentioned will be covered under the fund. spirometer & carbon monoxide monitor estimated at 2000 dollars per unit.

4) Materials & supplies: Nicotine patches in the intervention group will be estimated at 120 patches per participant for 16 weeks; 90 patches per participant in the control group for 12 weeks. Nicotine gums are estimated at 600 gums per participant in both the groups. Four saliva cotinine kits are required for each participant at 0, 3 months, 6 months, and 12 months. All the mentioned materials & supplies will be covered under the fund. Nicotine patch is estimated at 8 dollars per piece & nicotine gums at 0.35 dollars per piece. saliva cotinine test at 8 dollars per piece

5) Publication cost: This section will cover the cost of publication (proof reading, charges for color printing etc.) in high ranked journals, if necessary.

6) Subcontracts will be given to hire computer services such as software/mobile app development to record participant details.

5. Research group

Chethan Purushothama, MBBS, MD.as the Principal investigator of this proposal has several years of experience in tobacco control & related fields, He got his MD degree from Manipal university, India. Later he has worked as a Assistant professor in the Medical University of the Americas in the Caribbean, currently he is an assistant professor in the school of sciences & humanities, Nazarbayev university. During his rotations after MBBS he was involved in visiting primary & secondary heath centers & has helped many patients to quit smoking & chewing of tobacco (Gutka) which is rampant in India. This experience with tobacco cessation programs will add much knowledge to conduct the research.& help the population to stop smoking. Besides this Dr Chethan is involved in teaching for graduate & undergraduate students, he has two PhD students as GTA under him. He has published 13 journal & conference articles published in the field of Human Gross Anatomy, Human Anthropometry, Neurosurgery & clinical journals.

Byron Lawrence crapePhD MSPH, BSas the Co-principal investigator has several years of experience in tobacco Board worldwide. To his credit he has more than 50 journal publications in which 25 are peer reviewed & has several books published in his name in the field of public health & epidemiology. He pursued his PhD at Johns Hopkins in Maryland, acting as a teaching assistant and later teaching a range of courses and guest lecturing. During his time at Hopkins, he established workshops to support graduate and medical students in biostatistics and epidemiology and founded the Health and Human Rights Group. He was a biostatistician for a range of major on-going cohort studies and clinical trials at Hopkins, including HIV/AIDS, addictions, program evaluations, and treatment protocols. He also worked on many dozens of smaller projects as a biostatistician and analytical epidemiologist. For his PhD he received a grant from NIH to conduct a study of the associations of weight cycling and type II diabetes (both hormonal processes) on postmenopausal breast cancer. Outside of these research and teaching activities on-location at Hopkins, Byron has also supported health service evaluations and taught various courses for Hopkins in Cambodia, Vietnam, Afghanistan and Spain. While in Baltimore he also worked for the State of Maryland in the AIDS Administration as a research statistician and as a TB Epidemiologist for the TB Division of the Maryland State Department of Health & Mental Hygiene. Following these positions, Byron worked with the WHO office for the region that included Cambodia, Vietnam, and the Philippines. On completion of that contract, he took a position in the technical unit of the regional headquarters of the World Health Organization (WHO) for the Western Hemisphere, based in Washington, DC. Responsible for a large variety of projects and technical assessments in this office, his responsibilities included coordination of major renovations on-location for national health information systems in Suriname, Trinidad and Tobago, Belize and other countries. Following these positions, Byron worked with the WHO office for the region that included Cambodia, Vietnam, and the Philippines. Leaving the regional WHO headquarters to work in a WHO field office in Caribbean Guyana, he taught a variety of workshops and courses to a variety of health professionals and students.With his high credentials & experience in working with different tobacco boards worldwide he will give an outstanding input to this project.

Valentina Stolyarova is a qualified psychiatrist, having worked at Karaganda Regional children's mental hospital and the Karaganda Regional mental hospital since 2006. Her work has focused on acute psychoses

and neuroses. She has also worked as a psychotherapist with patients and students and began as an instructor at Nazarbayev University School of Medicine in May 2017. Her research interests are extensive and includes Autism, Obsessive-Compulsive and Related disorders, Systemic family and marital psychotherapy, Erickson's hypnosis, Psychodrama and role playing with group and individual treatment sessions. She has completed projects relating to the prevention of the spread of social epidemics (drug, extremism, terrorism, involvement in criminal communities, destructive sects, gambling) in the Republic of Kazakhstan and family risk factors in the formation of neurotic, stress-related and somatoform disorders and "Diagnosing children with intellectual impairment and autism in Kazakhstan". With an excellent clinical background Valentina will be involved in counselling & treating the participants in the study.

Altynshash Jaxybayeva as a CO-Principal investigator, is the Head of neurology department at Astana medical university, Nur-Sultan, Kazakhstan. She has been involved in guiding students in the field of Neurology. She is involved in research activity in genetic diseases with neurological presentation like neuromuscular diseases, epileptic encephalopathy & neonatal neurology. She is a consultant at Paediatric neurology departments at UMC NRCMCH, Children hospital 2, 1, 3 and National Research Cardiac Centre. She has over 17 publications in which 12 are in English journals. with her extensive experience & research background she will counsel the participants in tobacco cessation.

PI and Co-PIs will be supervisors of young MPH/Graduate students. The MPH/Graduate students will learn about tobacco control & cessation programs which will help them to do further research in Kazakhstan. Team meetings will be held to share finding of different students. This will help the team to be more connected to each other, avoid duplication, learn each other's experience, and to help each other to achieve the goals of the main project.

6. Research environment and training for scientists

The study will involve experienced and young scientists, master and bachelor students of Nazarbayev University. Every member of the project will be involved in the Nicotine replacement therapy program. Scientific importance and the high productivity of the research will be achieved by the integration among the project members.

The project has well-defined objectives which will lead to the implementation of the Afterload nicotine replacement therapy. The equipment's & materials will have to be purchased under the grant funds. An area of 500 square feet will be required at the cardiac surgery research Centre for counselling & nicotine replacement treatment.

This research will compose of two groups, an experimental group & the control group. In both the groups nicotine patches & gums will be given. In the experimental group there will be preloading of nicotine patches. The following equipment will be used for research.

1) Portable spirometers
2) Carbon monoxide monitor
3) Sphygmomanometer
4) Weighing scale
5) Office furniture and computers

There will be young 3 MPH Graduates recruited in the program. The participation of young scientists in the project will allow them to integrate into the global scientific community.

7. Expected results

Scientific and social impact of the study results:

The study focuses on establishing high certainty evidence about the effectiveness of preloading combination nicotine replacement therapy on smoking cessation of adult population. With this outcome, the study shall help the medical practice to improve the cessation rates among tobacco smokers thereby reducing the costs incurred by the health systems. Future systematic reviews and meta- analysis shall develop high certainty evidence from this study which in turn helps the medical professionals to benefit the smokers' community through effective cessation prescriptions.

Since there no universally accepted therapy regimen for nicotine replacement therapy, the effectiveness of nicotine replacement therapy plays a crucial role in improving the quit rates among the smokers that may be a primary level of prevention of tobacco related diseases. This outcome is achievable only when research is conducted in the community using novel therapy regimens.

Expected results of this project will improve our understanding of Afterload of nicotine replacement therapy and will have significant contribution to further develop the study in the Adult population of Kazakhstan. We expect to gain knowledge and train young scientists in the following items:

1) Preload and standard nicotine replacement therapy
2) Counselling the participants to quit smoking.
3) Learn about the complications of smoking & diseases associated with it.

Also, the following outcomes are expected from this project:

- Publications of at least 5 articles in international journals with high impact factor.
- Participation and presentation in international conferences
- Training of young scientists, MPH /Graduate students in the research team.

8. References

The section shall specify most relevant publications/research conducted on the topic of the Project.

1. Ministry of Healthcare and Social Development of the Republic of Kazakhstan. Global Adult Tobacco Survey (Gats) of the Republic of Kazakhstan, 2014 country report. [cited on 13 May 2021]. p11. Available from https://www.who.int/tobacco/surveillance/survey/gats/kaz_countryreport_en.pdf?ua=1

2. Drope J, Schluger N, Cahn Z, Drope J, Hamill S, Islami F, et.al. The Tobacco Atlas. Atlanta: American Cancer Society and Vital Strategies.2018 [cited on 29 April 2021]. Available from:https://tobaccoatlas.org/country/kazakhstan/#:~:text=Tobacco%20harms%20the%20health%2C%20the,to%20use%20tobacco%20each%20day.

3. Hays JT, Croghan IT, Baker CL, Cappelleri JC, Bushmakin AG. Changes in health-related quality of life with smoking cessation treatment. Eur J Public Health. 2012;22(2):224-229.

4. Wang Q, Mati K. Intention to Quit among Smokers in Kazakhstan: Data from 2014 Global Adult Tobacco Survey. J Epidemiol Glob Health. 2019; 9(1): 23-28.

5. Levy DT, Levy J, Mauer-Stender K. Potential impact of strong tobacco-control policies in 11 newly independent states. Cent Eur J Public Health. 2019;27(2):115-126.

6. Giulietti F, Filipponi A, Rosettani G, Giordano P, Lacoacci C, Spannella F et.al. Pharmacological Approach to Smoking Cessation: An Updated Review for Daily Clinical Practice. High Blood Pres Cardiovasc Prev. 2020; 27(5): 349–362.

7. Lindson N, Chepkin SC, Ye W, Fanshawe TR, Bullen C, Hartmann-Boyce J. Different doses, durations and modes of delivery of nicotine replacement therapy for smoking cessation. Cochrane Database of Systematic Reviews 2019, Issue 4. Art. No.: CD013308. DOI: 10.1002/14651858.CD013308.

8. Herman A I, Sofuoglu M. Medications to Treat Addictions: Nicotine Replacement. Interventions for Addiction. Academic Press. 2013; 337-343.

9. Flowers, Lee M. Nicotine Replacement Therapy. The American Journal of Psychiatry Residents' Journal. 2016; 11 (6): 4-7.

10. The preloading investigators. Effects on abstinence of nicotine patch treatment before quitting smoking: parallel, two arm, pragmatic randomised trial. BMJ 2018; 361.

11. Prochaska JJ. Nicotine Replacement Therapy as a Maintenance Treatment. JAMA. 2015;314(7):718-719.

12. Chai, W., Zou, G., Shi, J. et al. Evaluation of the effectiveness of a WHO-5A model based comprehensive tobacco control program among migrant workers in Guangdong, China: a pilot study. BMC Public Health. 2018: 18 (296).

13. Dhavan P, Bassi S, Stigler MH, Arora M, Gupta VK, Perry CL et. Al. Using salivary cotinine to validate self-reports of tobacco use by Indian youth living in low-income neighborhoods. Asian Pac J Cancer Prev. 2011;12(10):2551-4. PMID: 22320954; PMCID: PMC3310162.

14. Vasthare R, Kumar S, Arron LY. Carbon monoxide breath analyzers and its role in tobacco cessation: A narrative review of literature. J Int Oral Health. 2018 [cited 2021 May 13]; 10:71-6. Available from: https://www.jioh.org/text.asp?2018/10/2/71/230862

15. Rose JE, Behm FM, Westman EC. Nicotine-mecamylamine treatment for smoking cessation: the role of pre-cessation therapy. Experimental and Clinical Psychopharmacology. 1998; 6 (3):331-43.

16. Hymowitz N, Cummings KM, Hyland A, Lynn WR, Pechacek TF, Hartwell TD. Predictors of smoking cessation in a cohort of adult smokers followed for five years. Tob Control. 1997; 6 (2): 57-62.

9. Ongoing projects

Chethan Purushothama the privipal investigator is a Co-principal investigator for the study: Affordable Robot Assisted Gait Rehabilitation for Stroke Survivors.
